# Evaluation of higher cognitive functions following posterior quadrant disconnection in the non-dominant hemisphere: a Case Report

**DOI:** 10.3389/fnhum.2025.1569673

**Published:** 2025-07-17

**Authors:** Noeru Kawase, Shunsuke Nakae, Masanobu Kumon, Motoharu Hayakawa, Chiaki Shinzato, Yuriko Sato, Takehiro Uda, Takumi Mitsuhashi, Masaki Fukunaga, Shigeo Ohba, Yuichi Hirose

**Affiliations:** ^1^Department of Neurosurgery, School of Medicine, Fujita Health University Hospital, Toyoake, Aichi, Japan; ^2^Rehabilitation Complex, Fujita Health University, Toyoake, Aichi, Japan; ^3^Department of Neurosurgery, Osaka Metropolitan University, Osaka, Japan; ^4^Department of Neurosurgery, Juntendo University, Tokyo, Japan; ^5^Division of Cerebral Integration, National Institute for Physiological Sciences, Okazaki, Aichi, Japan

**Keywords:** posterior quadrant disconnection, drug-resistant epilepsy, non-dominant hemisphere, higher cognitive function, hemispatial neglect, visuospatial cognitive functions

## Abstract

Posterior Quadrant Disconnection is a surgical technique designed to suppress seizure propagation while preserving motor and sensory functions in patients with drug-resistant epilepsy. Although seizure outcomes following this procedure have been reported, detailed evaluations of its impact on higher cognitive functions remain limited. This study aimed to assess the long-term seizure and cognitive outcomes following PQD in the non-dominant hemisphere, thereby evaluating the efficacy and safety of the procedure. In this case, the patient with drug-resistant epilepsy underwent preoperative evaluation using stereo electroencephalography (SEEG) to identify seizure onset zones and functional mapping related to visuospatial cognition. Following this assessment, PQD was performed. Postoperative outcomes were monitored over a 2-years period, focusing on seizure control and higher cognitive function. The patient achieved Engel class I status postoperatively, indicating complete seizure cessation. While transient hemispatial neglect was observed immediately after surgery, gradual improvement was noted over time. Furthermore, visual memory and cognitive functions showed a tendency to improve, and there were no significant declines in facial recognition or scene recognition abilities. These findings suggest that PQD can effectively improve seizure outcomes while minimizing long-term impacts on cognitive functions. This case highlights the potential of PQD to offer substantial seizure control with limited permanent effects on higher cognitive functions. By providing valuable insights into the safety and efficacy of PQD in the non-dominant hemisphere, this study underscores its viability as a treatment option for selected cases of drug-resistant epilepsy.

## Introduction

Posterior quadrant disconnection is a surgical procedure intended to interrupt the propagation of seizures originating from extensive regions of the temporal, parietal, and occipital lobes in patients with drug-resistant epilepsy (Barrit et al., 2022). Previous studies have provided evidence supporting the effectiveness of this intervention in controlling seizures while preserving motor and sensory functions (Markosian et al., 2021; Davis et al., 2012; Daniel et al., 2007).

Posterior quadrant disconnection involves the disconnection of the parietal, temporal, and occipital lobes, which is expected to lead to postoperative effects on higher cognitive functions. Disconnection in the non-dominant hemisphere may lead to visuospatial cognitive deficits due to disruption of the visual pathways. Specifically, interruption of the dorsal-ventral pathway may result in hemispatial neglect and hemisomatognosia (Heilman et al., 1985; Bisiach and Luzzatti, 1978). Furthermore, disruption of the dorsal pathway can lead to impairments in spatial navigation, while disruption of the ventral pathway may cause prosopagnosia and associated dressing apraxia (Zhang et al., 2006; Takahashi et al., 1997; Katayama et al., 1999; Barton, 2008; Brain, 1941). In addition, extensive disconnection in the right hemisphere may result in deficits in emotional cognition (Blake, 2003).

Despite the potential benefits of PQD, the procedure is only performed in a limited number of specialized centers, leading to a scarcity of reported cases. To date, the PQD case series with the largest number of reports is that by Rizzi et al. (2019), though there have been no detailed reports on the postoperative effects on higher cognitive functions, and the impact of PQD on higher brain functions remains insufficiently explored.

In this case, we conducted brain functional mapping (passive functional mapping) for visuospatial cognition during SEEG insertion and assessed postoperative seizure outcomes and long-term higher cognitive functions. This report aims to evaluate the effectiveness and safety of PQD.

## Case presentation

A male patient in his 30s, naturally left-handed but with a history of hand dominance transfer, presented with drug-resistant epilepsy. Although details of perinatal complications were unknown, he had a history of febrile seizures at the age of 2. No developmental delays had been reported. At approximately 18 years of age, he developed epilepsy and began treatment with antiseizure medications, but his condition progressed to drug-resistant epilepsy. He experienced focal impaired awareness seizures (FIAS) 3–4 times per month and was referred to our institution. At the time of referral, his medication regimen included 600 mg of carbamazepine, 2000 mg of levetiracetam, and 200 mg of lacosamide.

**FIGURE 1:**
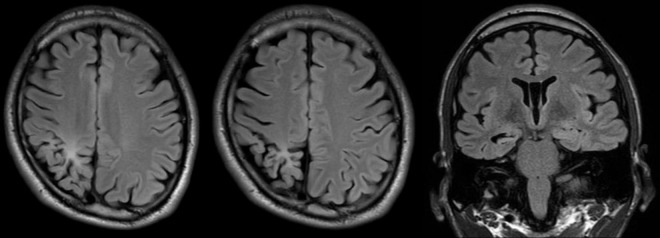
Preoperative Magnetic resonance imaging Fluid-attenuated inversion recovery (MRI FLAIR) image of the patient. Hyperintensity is observed from the right parietal lobe to the occipital lobe. Right hippocampal sclerosis is noted, and there is generalized atrophy of the right hemisphere.

The seizures were characterized by nausea as an aura and progressed to tonic-clonic seizures starting with motion arrest. Seizures predominantly occurred during the daytime and were often triggered while using the toilet. In recent years, he began experiencing upper abdominal discomfort as an aura. On physical examination, partial left homonymous hemianopia had already been identified by an ophthalmologist. Magnetic resonance imaging (MRI) revealed Fluid-attenuated Inversion Recovery (FLAIR) hyperintensity from the right parietal to occipital lobes, right hippocampal sclerosis, and overall atrophy of the right cerebral hemisphere (Figure 1). Interictal electroencephalogram (EEG) demonstrated clear lateral asymmetry, with overall low-amplitude signals in the right hemisphere, including baseline rhythms, but no obvious epileptiform discharges were observed.

Surgery was considered as a treatment option, and long-term video EEG monitoring was performed. Although several seizures were captured, the seizure onset zone could not be identified on EEG. After monitoring, a neuropsychological evaluation was conducted. Wechsler Adult Intelligence Scale – Fourth Edition (WAIS-IV) results were as follows: full-scale intelligence quotient 70, verbal comprehension index 74, perceptual reasoning index 85, working memory index 82, and processing speed index 63. Wechsler Memory Scales – Revised (WMS-R) scores showed verbal memory 62, visual memory 85, general memory 65, attention/concentration 94, and delayed recall below 50 (Table 1). The Wada test confirmed left hemisphere dominance. Since the epileptogenic zone could not be definitively localized, stereo electroencephalography (SEEG) was planned.

**TABLE 1:** Neuropsychological evaluation of the patient.

Test/assessment	Pre PQD	Post	Post 6 months	Post 1 year	Post 2 years
BIT	140/146	100/146	135/146	140/146	142/146
**WAIS-IV**					
FSIQ	70		69	76	70
VCI	74		77	85	79
PRI	85		82	84	84
WMI	82		74	79	76
PSI	63		66	71	60
**WMS-R**					
Verbal memory	62		66	88	92
Visual memory	85		90	108	108
General memory	65		77	93	96
Attention/concentration	94		92	85	87
Delayed recall	Below 50		71	99	105
Facial recognition	*				**
Topographical agnosia	50/52		49/52	50/52	50/52

BIT, Behavioral Inattention Test; WAIS-IV, Wechsler Adult Intelligence Scale – Fourth Edition; FSIQ, full-scale intelligence quotient; VCI, verbal comprehension index; PRI, perceptual reasoning index; WMI, working memory index; PSI, processing speed index; WMS-R, Wechsler Memory Scales–Revised.

*Answered according to his knowledge,

**Exactly the same answers as Pre PQD.

**FIGURE 2:**
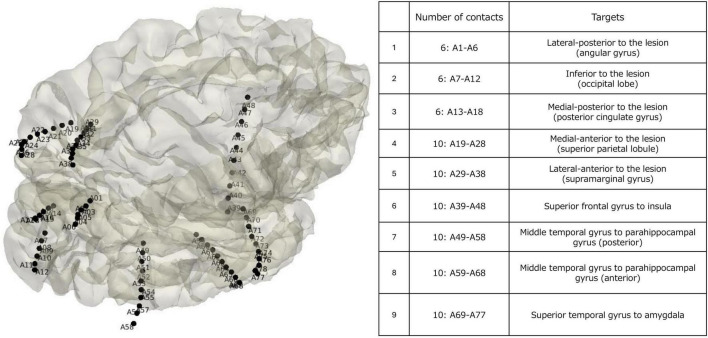
3D rendering and projected stereo electroencephalography (SEEG) electrodes of the patient. The numbers in the figure represent the electrode numbers. A total of nine electrodes were inserted into five locations, including the precentral gyrus, amygdala, parahippocampal gyrus, and posterior parahippocampal gyrus, to surround the hyperintense area observed on the Magnetic resonance imaging Fluid-attenuated inversion recovery (MRI FLAIR) image. The seizure onset zones for each captured seizure were identified at the following electrodes: A15, A43, A60, A63, B5, and B8.

Stereo electroencephalography was performed, and a total of nine electrodes (78 contacts) were implanted, including five surrounding the FLAIR hyperintense region and four targeting the insula, amygdala, anterior parahippocampal gyrus, and posterior parahippocampal gyrus (Figure 2). During 7 days of monitoring, four FIASs were captured with seizure onset zones originating from three distinct contacts implanted in the insular cortex, parahippocampal gyrus, and superior parietal lobule. The FIASs were characterized by unresponsive behavioral arrest with no recollection by the patient, sometimes progressing to generalized convulsions. Including subclinical seizures and focal aware seizures (FAS), additional seizure onset zones were identified in the amygdala; the FASs were accompanied by subjective nausea. As a result, six contacts with seizure onset activity were identified. The epilepsy network was thought to propagate via the ventral pathway, and non-dominant hemisphere PQD combined with insulectomy and amygdala resection was planned.

After the completion of seizure monitoring, passive functional mapping was performed using Corti Q (g.tec Medical Engineering GmbH, Austria; version V1.12.01), while the nine deep electrodes remained in place. The task consisted of alternating 5-s rest and 5-s task periods, repeated 100 times. Event-related high-gamma activity (60–140 Hz) was analyzed using time-frequency decomposition with Morlet wavelets (FieldTrip toolbox). Raw data exported from CortiQ were segmented and baseline-corrected using EEGLAB v2019.1, and a bipolar montage was applied. Responses were considered significant if the averaged power spectra exceeded a Z-score of 2.0 from baseline (Mitsuhashi et al., 2023). Tasks included facial recognition, landmark recognition, color Stroop, visual memory, and emotional recognition. The facial recognition task involved a Famous Faces Test, which utilized photos of celebrities from the patient’s favorite TV genre, interspersed with unrelated faces. This approach allowed for an ecologically valid assessment of face recognition, simulating real-life conditions without relying on standardized tests. Although the task involves elements of semantic memory, it effectively demonstrated the patient’s preserved ability to recognize familiar individuals. The landmark recognition task included photos of the patient’s neighborhood and images of other locations. The visual memory task was based on previous studies (Kunii et al., 2014; Kapeller et al., 2018). Specific activity was observed propagating from the occipital lobe through the fusiform gyrus to the parahippocampal gyrus during the facial and emotional recognition tasks.

**FIGURE 3:**
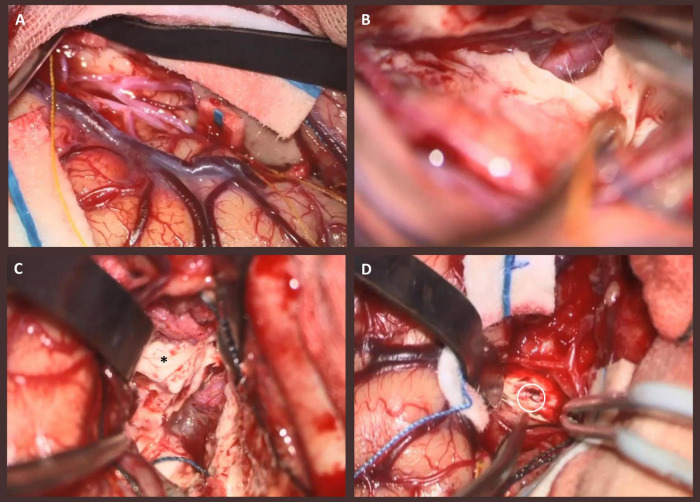
Intraoperative photographs of the patient. **(A)** After sufficient dissection of the Sylvian fissure to its distal portion, the surface of the insular cortex becomes visible, with the M2 segment and its branching vessels clearly observed. **(B)** Medial disconnection of the temporal lobe. After creating a disconnection plane from the uncus to the head of the hippocampus, the ambient cistern was identified, and the disconnection was extended posteriorly along the trajectory of the posterior cerebral artery. **(C)** Inferior disconnection of the parietal lobe. The cingulate gyrus was disconnected, and the disconnection plane was extended along the falco-tentorial junction to connect the medial surfaces of the temporal and parietal lobes. The posterior portion of the disconnected hippocampus is indicated with an asterisk. **(D)** Photograph of the insulectomy. The contact within the seizure onset zone (SOZ) is indicated with a circle.

**FIGURE 4:**
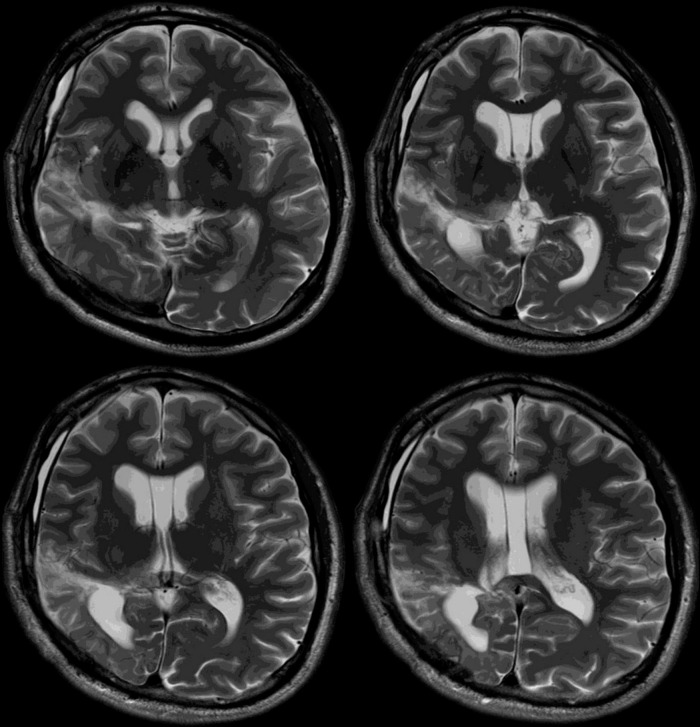
Magnetic resonance imaging (MRI) T2 image of the patient after Posterior quadrant disconnection (PQD) and insulectomy. Only the dissection plane is observed.

Two weeks after SEEG, PQD and insulectomy were performed on the non-dominant hemisphere (Figure 3). A right frontotemporal craniotomy was performed. After sufficient dissection of the Sylvian fissure to its distal portion, the procedure was initiated with disconnection of the temporal lobe. The inferior periinsular sulcus was incised to reach the inferior horn of the lateral ventricle. The disconnection line was then extended anteriorly along the temporal lobe to its anterior tip. A portion of the amygdala was resected, the inferior choroidal point was identified, and from there, the hippocampal head and uncus were disconnected anteriorly, reaching the ambient cistern. While carefully identifying the posterior cerebral artery, medial temporal disconnection was extended posteriorly, creating a disconnection plane from the inferior horn to the atrium of the lateral ventricle. Subsequently, parietal disconnection was initiated. The disconnection line on the parietal side was made to include the contacts located in the seizure onset zone on the side to be disconnected, by resecting part of the superior parietal lobule. Medial disconnection was carried out up to the falx cerebri. Upon reaching the body of the lateral ventricle, the medial wall of the ventricle was also disconnected, followed by disconnection of the cingulate gyrus. The disconnection was extended from the inferior edge of the falx to the tentorium, completing the procedure by transecting the fornix. An insulectomy was performed down to Contact A43, which corresponded to the SOZ in the insula, and the surrounding insular cortex was also resected as extensively as possible along the surface (Figure 4). Since partial left homonymous hemianopia was pre-existing, severe hemispatial neglect was not anticipated. However, significant hemispatial neglect symptoms were observed immediately after surgery with complete homonymous hemianopia due to the disconnection postoperatively. A marked decline in Behavioral Inattention Test (BIT) scores was observed postoperatively. The patient continued rehabilitation therapy and was discharged home 21 days after the PQD.

Post-discharge, the patient experienced transient anxiety, accompanied by episodes of excessive water intake. He received psychiatric care including pharmacological treatment, and the symptoms resolved within 5 months. Occasional episodes of wearing clothes inside out, suggestive of dressing apraxia, were reported during the first 6 months postoperatively but resolved within 1 year. No seizures occurred postoperatively, with seizure outcomes classified as Engel Class I. Higher cognitive functions were monitored for 2 years after surgery. BIT scores, while significantly reduced postoperatively, gradually improved. WMS-R scores demonstrated notable improvement, while facial and landmark recognition tasks showed no differences compared to preoperative performance. However, WAIS-IV scores remained unchanged, showing no improvement during the follow-up period. In reporting this case, consent was obtained from the patient.

## Discussion

Posterior quadrant disconnection was performed on patients with drug-resistant epilepsy, in whom left hemisphere dominance was confirmed through the Wada test. The procedure resulted in seizure freedom, with no decline in higher cognitive functions observed during a 2-years long-term follow-up. A 2-years follow-up period was selected based on previous studies indicating that neuropsychological performance typically shows meaningful improvement and tends to stabilize within the first 1–2 years following epilepsy surgery (Baxendale et al., 2012). Previous studies on PQD, including the large case series by Rizzi et al. (2019), have primarily focused on seizure control and sensorimotor outcomes. This case provides novel insights by presenting a systematic postoperative neuropsychological assessment of higher-order visual cognitive functions, thereby expanding understanding of the functional impact of PQD. To the best of our knowledge, this is the first study to conduct such a detailed, long-term assessment of higher cognitive functions following PQD.

The non-dominant hemisphere is responsible for spatial information processing, and its impairment is often associated with hemispatial neglect. Lesions in the angular gyrus, supramarginal gyrus, and superior temporal gyrus of the non-dominant hemisphere have been identified as key regions responsible for this condition (Mort et al., 2003; Corbetta et al., 2005; Karnath et al., 2004). In this case, severe hemispatial neglect was observed immediately after PQD, with the patient failing to recognize the existence of their right side, leading to minimal movement of the affected side. The BIT score showed a significant decline postoperatively, but as shown in Table 1, this decline was transient, and the score subsequently returned to preoperative levels. The BIT is a standardized test designed to assess various symptoms of hemispatial neglect in a manner relevant to daily life (Halligan et al., 1991). These findings suggest that the symptoms of hemispatial neglect improved over time, the patient adapted to the condition, or both mechanisms contributed to recovery. Previous studies on hemispatial neglect in stroke patients have reported significant improvements in spatial neglect symptoms during the acute phase, particularly within the first 3 months, with neurological recovery largely dependent on the passage of time (Nijboer et al., 2013). In this case, recovery from hemispatial neglect following PQD may support the potential roles of neuroplasticity and compensatory mechanisms, suggesting that permanent neglect symptoms were avoided. While hemispatial neglect is generally thought to result from deficits in spatial information processing and attention (Mesulam, 1999), visual field defects reflect visual impairment due to the loss of field information. Moreover, hemispatial neglect has been reported to manifest in auditory and body-centered spatial domains, not solely in the visual domain (Bisiach et al., 2001). In this case, despite the progression from partial to complete left homonymous hemianopia postoperatively, hemispatial neglect symptoms improved to preoperative levels. This observation supports that hemispatial neglect is not solely dependent on visual function but also involves broader spatial and attentional processing mechanisms.

Facial recognition is primarily associated with the right temporal lobe, particularly the parahippocampal gyrus and, more specifically, the right fusiform gyrus. However, many reported cases of prosopagnosia involve bilateral lesions. Similarly, landmark recognition is thought to rely on the posterior region of the splenium of the corpus callosum in the right hemisphere, although cases of topographical agnosia due to left-sided lesions have also been reported (Osawa et al., 2008). In this case, evaluations of facial and landmark recognition conducted post-PQD revealed no significant differences in task performance compared to preoperative assessments. From these findings, two possibilities can be inferred. First, both facial and landmark recognition deficits are more likely to occur with bilateral rather than unilateral brain lesions. Second, individual variability exists in the lateralization of brain functions, including facial and landmark recognition. This patient had experienced epilepsy since adolescence, and it is conceivable that during this time, as the functional capacity of the right hemisphere declined, the left hemisphere compensated for functions typically performed by the right hemisphere. While the ventral pathway in the right hemisphere is commonly implicated in facial recognition, interactions with the ventral pathway in the left hemisphere, responsible for word recognition, have also been reported (Behrmann and Plaut, 2014). This suggests that after PQD in the right hemisphere, the left hemisphere may have compensated for facial recognition tasks. Overall, this case provides a possible indication of the involvement of both hemispheres in facial and landmark recognition, highlighting the complexity and adaptability of these cognitive processes.

Previous studies have reported functional assessments following cerebrovascular diseases (Potter et al., 2021); however, the differences in pathophysiology between cerebrovascular diseases, which are characterized by vascular territory-dependent acute onset, and epilepsy surgeries, which involve chronic progression and carefully planned disconnections along gyri and functional regions, suggest that the outcomes of higher cognitive function assessments would also differ. Cerebrovascular lesions depend on vascular territories and often result in extensive damage spanning multiple gyri. This extensive damage often leads to simultaneous impairments in corresponding functions, making it challenging to examine detailed anatomical and pathological correlations in long-term survivors or to identify precise functional localization in each case (Decroix et al., 1986; Sugimoto et al., 2012). In contrast, epilepsy surgery involves tailored disconnections with consideration of the gyri, allowing for a clearer correlation between anatomy and function. This approach enables a more precise understanding of functional localization and brain networks (Umaba et al., 2018). Additionally, while indirect evaluations using functional MRI (fMRI) rely on changes in cerebral blood flow during hypothetical tasks, these results can be influenced by the patient’s systemic and mental state (Silva et al., 2017). Epilepsy surgery, on the other hand, provides direct assessments through surgical disconnections, allowing for more reliable post-surgical evaluations of higher cognitive functions. Since epilepsy progresses chronically, compensatory mechanisms may develop within the brain, in addition to individual differences in brain function. By conducting preoperative functional mapping, it becomes possible to account for these individual differences and compensatory mechanisms, enabling targeted disconnections. Postoperative evaluations of higher cognitive functions can then provide detailed insights into the effects of surgical intervention on brain function. This approach highlights the utility of epilepsy surgery not only as a treatment but also as a method for advancing our understanding of functional brain organization.

In this case, passive functional mapping for visuospatial cognitive functions was performed during SEEG monitoring. Specific activity propagating from the occipital lobe through the fusiform gyrus to the parahippocampal gyrus was observed during facial recognition and emotional recognition tasks. However, the postoperative evaluation of higher cognitive functions following PQD revealed no changes in task responses. While reports have demonstrated the utility of functional mapping for predicting surgical outcomes in language functions (Kanaya et al., 2021). However, it remains unclear whether visuospatial cognitive functions are lateralized or bilateral. Therefore, the utility of passive functional mapping in predicting postoperative visuospatial cognitive outcomes requires further investigation.

In this case, a clear improvement was observed in the WMS-R scores, whereas the WAIS-IV scores showed minimal change even at 2 years postoperatively. One possible explanation is that differences in the constituent components of the WAIS-IV and WMS-R influenced these outcomes. Previous studies have indicated that improvement in intelligence quotient requires long-term neuro-network reorganization, with WAIS-IV improvements typically manifesting after extended follow-up periods of 5 years or more (Skirrow et al., 2011). In contrast, the WMS-R specifically evaluates memory function and is more sensitive to compensatory mechanisms in temporal lobe memory circuits, with some reports demonstrating improvement within approximately 1 year after surgery (Sugano et al., 2022).

This study has several limitations. First, it is the only case to date that provides a detailed evaluation of higher brain functions following PQD in the non-dominant hemisphere. Additional case studies are required to comprehensively assess brain function after PQD in the non-dominant hemisphere. Second, considering the invasiveness of the procedure, functional mapping was performed only on the non-dominant right hemisphere, without mapping the left hemisphere. Conducting functional mapping of the left hemisphere would help clarify whether higher cognitive functions are lateralized or involve both hemispheres. These findings emphasize the need for further research to better understand the role of hemispheric contributions and the potential applications of passive functional mapping in predicting surgical outcomes for visuospatial cognitive functions.

Posterior quadrant disconnection was performed on the non-dominant hemisphere, and higher cognitive functions were meticulously evaluated and analyzed over a 2-years postoperative period. This evaluation offers valuable information for understanding the impact of PQD on higher cognitive functions. While the patient’s preoperative partial left homonymous hemianopia progressed to complete hemianopia, their higher cognitive functions were not significantly impaired. These findings suggest that PQD targeting the extensive epileptic zone in the non-dominant hemisphere can be effective in improving seizure outcomes and functional prognosis, at least in this particular case.

## Conclusion

This study conducted a longitudinal evaluation of higher cognitive functions related to visuospatial cognition for 2 years following extensive disconnection of the non-dominant hemisphere through PQD. PQD in the non-dominant hemisphere may contribute to improved seizure outcomes and can potentially be performed without causing major impairment of higher cognitive functions. This study is important in that it provides a long-term assessment of higher cognitive functions of the epilepsy patient after disconnection of the temporal, occipital, and parietal lobes in the non-dominant hemisphere. Further studies are needed to determine whether these results can be generalized.

## Data Availability

The raw data supporting the conclusions of this article will be made available by the authors, without undue reservation.
